# The Boost of Toluene Capture in UiO-66 Triggered by Structural Defects or Air Humidity

**DOI:** 10.1021/acs.jpclett.3c00858

**Published:** 2023-06-13

**Authors:** Gabriela Jajko, Juan José Gutiérrez Sevillano, Sofia Calero, Wacław Makowski, Paweł Kozyra

**Affiliations:** † Faculty of Chemistry, Jagiellonian University in Kraków, Gronostajowa 2, 30-387 Kraków, Poland; ‡ Doctoral School of Exact and Natural Sciences, Jagiellonian University in Kraków, Łojasiewicza 11, 30-348 Kraków, Poland; § Department of Physical, Chemical and Natural Systems, Universidad Pablo de Olavide, Ctra. Utrera Km. 1, Seville ES-41013, Spain; ∥ Materials Simulation and Modelling, Department of Applied Physics, Eindhoven University of Technology, 5600 MB Eindhoven, The Netherlands

## Abstract

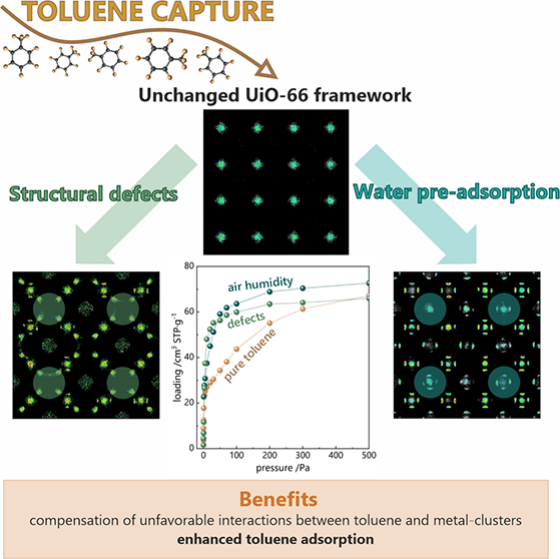

This work aimed to investigate the adsorption of toluene in UiO-66 materials. Toluene is a volatile, aromatic organic molecule that is recognized as the main component of VOCs. These compounds are harmful to the environment as well as to living organisms. One of the materials that allows the capture of toluene is the UiO-66. A satisfactory representation of the calculated isotherm steep front and sorption capacity compared to the experiment was obtained by reducing the force field σ parameter by 5% and increasing ε by 5%. Average occupation profiles, which are projections of the positions of molecules during pressure increase, as well as RDFs, which are designed to determine the distance of the center of mass of the toluene molecule from organic linkers and metal clusters, respectively, made it possible to explain the mechanism of toluene adsorption on the UiO-66 material.

VOCs are volatile or low-boiling organic substances that belong to the gaseous air pollutants.^1^ Examples of such relationships include but are not limited to^2−4^ aliphatic and aromatic hydrocarbons and hydrocarbon derivatives, alcohols, esters, and compounds that contain sulfur or nitrogen in their composition. VOCs can be formed naturally, e.g. through volcanic eruptions, and anthropogenically, through energy production or the chemical industry. They are harmful to the environment as they have a negative effect on woody vegetation, especially conifers. Moreover, due to their mutagenic properties, they play a significant role in the ever-increasing incidence of neoplastic diseases of the respiratory system.^5−7^ There are many ways to remove VOCs from the atmospheric air, among others: a method of thermal, catalytic, or biological oxidation;^8−12^ a condensation method;^13,14^ a membrane method;^15,16^ and an adsorption method.^17−19^ The adsorption method is based on the capture of harmful substances from the gas phase through the contact of polluted air with the surface of the adsorbent. The presence of water vapor in the adsorption stream has a detrimental effect on the performance of the adsorbents. This is because water vapor can compete with VOCs for adsorption sites, reducing the adsorbent’s ability to adsorb VOCs, especially at high RH.^20^ Such adsorbents include, for example, MOF-177. The research of Yang et al.^21^ was aimed at examining the adsorption of volatile organic compounds and the influence of humidity on their adsorption in the air. It has been proven that MOF-177, due to its large surface area and pore volume, can be an adsorbent for removing VOC particles from the air, especially those that are characterized by small dimensions. It was also found that the tested material showed a greater ability to adsorb at relatively high humidity. Comparing the MOF-177 material with active carbon under high humidity conditions, it was observed that the damping of adsorption in activated carbon was significantly greater than in the tested material. Nevertheless, MOF-177 should not be exposed to air of high humidity for a long time, moreover, the gas should be predried in order to inhibit competitive water adsorption and, consequently, decomposition of the MOF-177 skeleton.

UiO-66 material,^22^ unlike MOF-177, is highly resistant to contact with water vapor. It consists of metallic Zr_6_O_4_(OH)_4_ clusters in which a zirconium ion is present, and terephthalic acid (BDC) linkers. This material consists of two types of cages: tetrahedral (7.5 Å) and octahedral (12 Å), with 6 Å pore slits. The cages differ from each other in their position in relation to the metallic clusters, and thus in the orientation of the linkers to the interior. As a consequence, it affects, for example, the preferential adsorption of CO_2_^23^ or hydrocarbons, due to the stronger interaction between aromatic rings in tetrahedral cages. Nevertheless, some molecules, such as water or alcohols, prefer sorption in octahedral cages due to electrostatic interactions related to their polarity.^24^

In this work, we investigate the adsorption of toluene on UiO-66 material containing structural defects. For this purpose, the Monte Carlo method was used, using the RASPA code^25,26^ and an appropriately modified force field. After fitting the toluene adsorption isotherm on UiO-66 material, it was shown that the adsorbate accumulates within the organic linkers in tetrahedral cages. This is due to the orientation of the organic linkers in the aforementioned cages, which consequently results in better ring–ring interactions. The exception is high-pressure conditions, where toluene begins to fill also the spaces around the metal oxide clusters in octahedral cages.

Toluene adsorption isotherms were measured in three UiO-66 samples synthesized at different temperatures. It is known from previous studies that the lower the synthesis temperature, the more missing linker defects in the structure.^24^ So the UiO-66_100 sample contains the largest number of defects, while UiO-66_220 is considered defect-free. Having the toluene isotherm in linear scale (Figure 1, inset) shows that sorption occurs at very low pressures, with the adsorption steep front around 3 Pa (*p*/*p*_0_ ≈ 0.001) for all the samples. The shape of the isotherm may be classified as the IUPAC type II. The sorption capacity at the highest pressure (*p*/*p*_0_ ≈ 1) is around 118 cm^3^ STP/g for UiO-66_220, 137 cm^3^ STP/g for UiO-66_160, and 219 cm^3^ STP/g for UiO-66_100.

**Figure 1:**
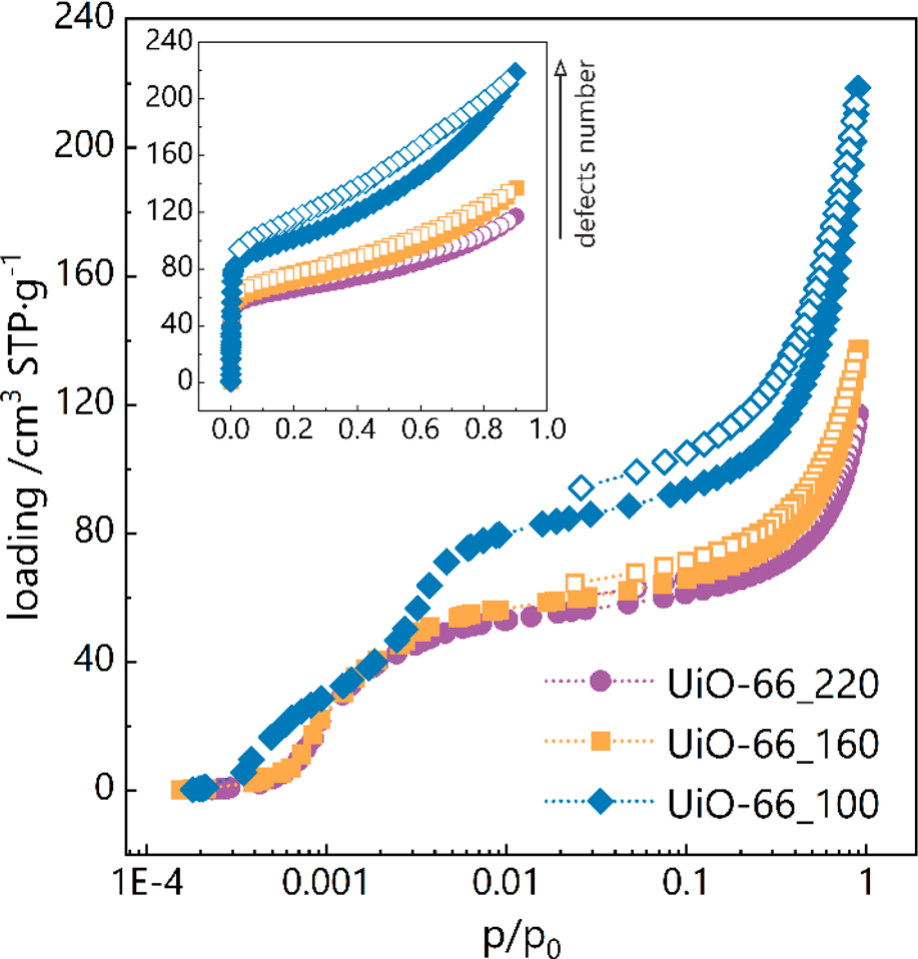
Toluene adsorption isotherms in UiO-66 samples with different content of defects in semilogarithmic scale, measured in 300 K. Inset shows isotherms in linear scale. Closed symbols stand for adsorption and open for desorption.

Considering the semilogarithmic scale of the isotherm (Figure 1), one can notice a different shape in the low-pressure range for the UiO-66_100 sample, containing the most structural defects. This indicates that microporosity is affected by the number of defects. Compared to nitrogen isotherms,^24^ stronger adsorption at low pressures is visible, indicating a specific interaction of toluene with the UiO-66 framework. More information about the locations of toluene will bring the analysis of Average Occupation Density Profiles (*vide infra*).

To gain insight into the adsorption mechanism, we performed Monte Carlo simulations. Literature force field parameters from Castillo et al.^27^ were not able to reproduce the experimental isotherm for a defect-free sample, so it was necessary to refine the force field parameters. Nonbonded interactions between guest molecules and the framework were modeled using a Lennard-Jones and Coulombic potential:
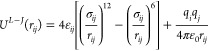
1where *r*_*ij*_ is a distance between *i* and *j* atoms, and *q*_*i*_ and *q*_*j*_ are atom charges, which were not changed. For each UiO-66_0 framework atom and toluene (pseudo)atoms

ε and σ values from Table 2 were used, which were mixed using Lorentz–Berthelot rules. Considering the underestimation of the interactions for toluene adsorption, the interactions between (pseudo)atoms and atoms of the UiO-66 framework were appropriately modified using the Lorentz–Berthelot mixing rules (reduction of σ interactions by 5% and increase of interactions ε by 5%). The modified parameters are summarized in Table 1. Original and modified calculated isotherm for defect-free material may be found in Figure S1 in the Supporting Information.

**Table 1:** Modified Lorentz–Berthelot Mixing Rules for Toluene Adsorption in UiO-66

	literature force field parameters		modified force field parameters	
type of interaction	ε/*k*_B_ (K)	σ (Å)	ε/*k*_B_ (K)	σ (Å)
CH_3-toluene_–C	63.973	3.637	67.171	3.455
C_–toluene_–C	41.068	3.512	43.121	3.336
H_–toluene_–C	26.820	2.947	28.161	2.799
CH_3-toluene_–H	25.576	3.323	26.855	3.157
C_–toluene_–H	16.419	3.198	17.240	3.038
H_–toluene_– H	10.723	2.633	11.259	2.502
CH_3-toluene_–Zr	54.489	3.292	57.214	3.127
C_–toluene_–Zr	34.980	3.167	36.729	3.008
H_–toluene_–Zr	22.845	2.602	23.987	2.472
CH_3-toluene_–O	64.193	3.417	67.403	3.246
C_–toluene_–O	41.209	3.292	43.270	3.127
H_–toluene_–O	26.913	2.727	28.258	2.590

The next step in the research was to explain the mechanism of toluene adsorption in the pores of UiO-66 material. For this purpose, Average Occupation Density Profiles (AOPs) were plotted, i.e., projections of the positions of molecules during increasing pressure. Based on AOPs in the *xy* direction (Figure 2a), it was possible to determine the preferential adsorption location region. From the aromatic structure of the toluene molecule, it can be predicted that it will prefer adsorption close to organic linkers. Indeed, these predictions were confirmed, and the phenomenon can already be seen from calculations for the pressure of 1 Pa: toluene fills the spaces in the tetrahedral cages. Linkers are oriented side into the tetrahedral cages, which enhances toluene to adsorb here due to more efficient ring–ring interaction. In increased pressure e.g. *ca.* 100 Pa, toluene fills less favorable spaces in the vicinity of metal–oxide clusters in octahedral cages. The interactions of toluene with a metal cluster are not preferential because of its oxide structure and its highly electrostatic environment. It is only at very high pressures that toluene has no place at the organic linkers, so it is forced to fill the space around the zirconium cluster.

**Figure 2:**
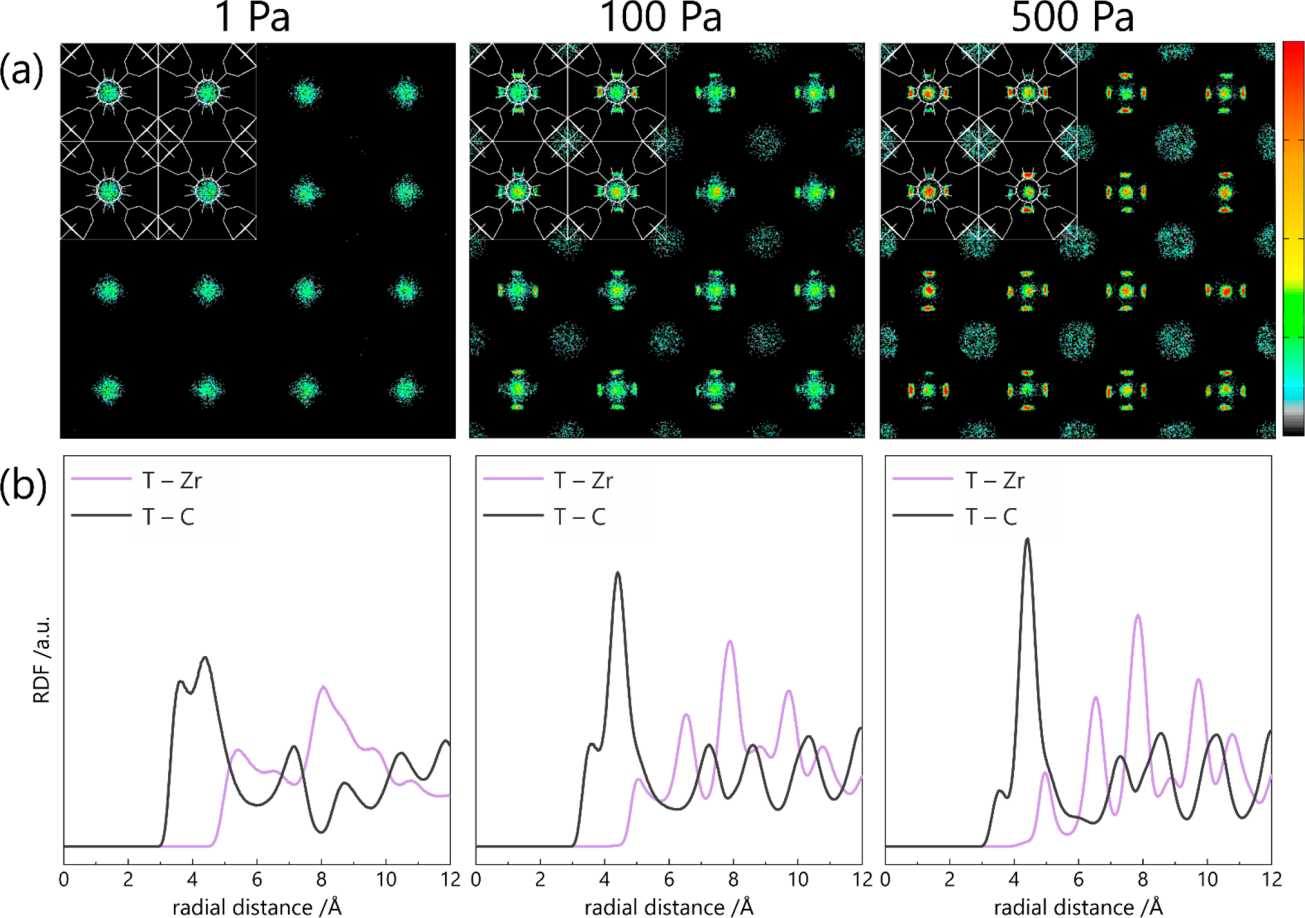
(a) Average occupation density profiles of toluene adsorption in UiO-66_0 structure in the xy direction for pressures of 1, 100, and 500 Pa, respectively. For easier interpretation, the UiO-66 structure model has been superimposed. (b) Radial distribution functions of toluene adsorption at a pressure of 1 (left), 100 (middle), and 500 (right) Pa.

To confirm the mechanism of preferential adsorption, Radial Distribution Functions (RDF) were modeled, which define distances of the center of mass of a toluene molecule from organic linkers (T–C) and metal clusters (T–Zr), respectively. When analyzing Figure 2b, it can be seen that the data is consistent with the conclusions drawn based on Average Occupation Profiles. At low pressure (1 Pa), toluene is at 3.5 and 4.3 Å from the organic linkers and 5.4 Å and more from the metallic cluster, which is precisely within the tetrahedral cage. The distance of 4.3 Å from organic linkers as the next neighbor excludes stacking of other toluene molecules, therefore, 0.8 Å supports surrounding the first molecule close to the metallic cluster. With increasing pressure, i.e., at 100 and 500 Pa, the distance between toluene and linkers does not change—they are still in the range of about 4 Å, which corresponds to the adsorption of further molecules (in other places of the 2 × 2 × 2 supercell), also within the tetrahedral cage. However, at higher pressures, despite the maximum of abundance of the distances between zirconium clusters and toluene at 5.4 Å, the shoulder at 5 Å appears, which is the beginning of toluene adsorption on metal clusters (in octahedral cages). Exactly this behavior was observed in Figure 2a when analyzing the Average Occupation Density Profiles.

The observed phenomenon is related to the aforementioned ring–ring interactions, which come to the fore because of the aromatic structure of both the guest molecule and organic linkers, and more specifically the π–π stacking. They occur precisely when two aromatic rings lie in planes parallel to each other (so-called face-to-face) or at an angle (so-called edge-to-face). In the case of the tested system, we are dealing with face-to-face, where the distance between them should be 3.3–3.7 Å,^28^ which is exactly the distance from the center of mass of toluene to the UiO-66 framework linkers. The interactions between aromatic rings are of dispersion nature, thus van der Waals equation, which is involved in our calculations, reproduced the well stabilizing effect of π–π stacking.

The UiO-66 material is known to contain structural defects, the concentration of which can be controlled by the synthesis temperature. Experimental studies (TG, EA, adsorption experiments) made it possible to determine the type of defects so formed.^24,29^ It was shown that they are vacancies of linkers–bulky fragments, significantly increasing the available void fraction. Therefore, the presence of defects is of great effect on adsorption properties. In previous studies, we demonstrated the effect of the presence of defects on the adsorption of water,^24^ polar and nonpolar molecules,^29^ and carbon dioxide capture.^23^ Based on the refined force field for toluene adsorption in ideal UiO-66, adsorption isotherms in defective materials were also calculated. As the introduced defects are the vacancies, the calculated isotherms should be significantly different in shape (Figure S2). As expected, the greatest change can be observed in the low-pressure range, in particular in the range from 0 to 300 Pa. As shown earlier in the analysis of AOPs, toluene molecules at low *p*/*p*_0_ adsorb on organic linkers, so the more space in this range (after removing several linkers in each cage), the more molecules are able to adsorb (face-to-face adsorption of one toluene molecule on another). When analyzing the AOPs (Figures S3–S6) for the defected structures we observe that the presence of additional adsorption spaces changes the adsorption of toluene. At low pressure (i.e., 1 Pa), the maps look exactly the same as for the nondefected sample.

To assess if water molecules change the adsorption of toluene, the effect of the presence of water on toluene adsorption was tested in two stages. First, we carried out water adsorption calculations at 300 K in the full *p*/*p*_0_ range, which corresponds to the preadsorbed water at a given relative humidity (RH). This step made it possible to determine the specific positions of water molecules in the unit cell at a variety given pressures. Next, we performed toluene adsorption calculations in the presence of a defined and controlled amount of water (100 and 300 molecules per unit cell, which corresponds to relative humidity around 20% and 60%, respectively). We performed preadsorption calculations, where we consider the water guest molecules, as a part of the host structure. To our surprise, RH equal to 20% not only did it not interfere with the adsorption of toluene, but also increased the adsorption in the range of low pressures even by about 45% (Figure 3c). It is important to note that the preadsorption effect of 100 water molecules per unit cell is even better than the introduction of 32 structural defects (Figure S7).

**Figure 3:**
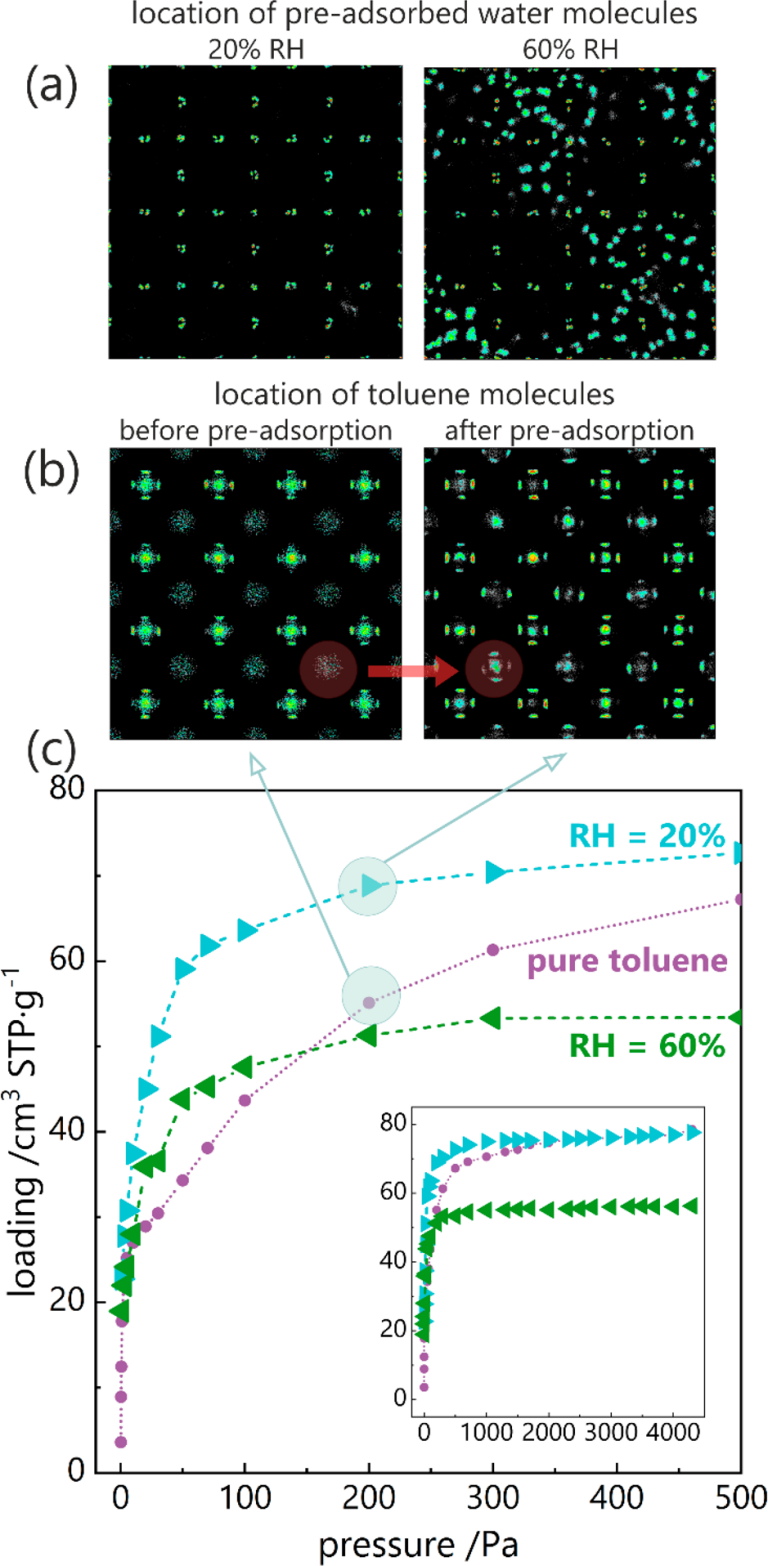
(a) Location of preadsorbed water molecules at relative humidity equal to 20% and 60%; (b) Location of toluene molecules before and after water preadsorption (20% RH), at 200 Pa. (c) Calculated pure toluene adsorption isotherm and toluene isotherms with preadsorbed water vapor (20% and 60% RH) at 300 K in UiO-66_0. Inset shows the isotherms in the full range.

Average Occupation Profiles show that pure toluene mainly absorb in the tetrahedral cages, as it was shown earlier (Figure 2). Water, at a relative humidity equal to 20%, absorb in the corners of octahedral cages, so around metal-clusters (Figure 3a). After preadsorption of water, toluene already at low pressure fills the spaces also in octahedral cages, which were previously avoided (Figure 3b). For RH = 60%, water molecules also begin to fill tetrahedral cages (Figure 3a), simultaneously occupying potential adsorption sites for toluene (as water is preadsorbed and treated as part of the host in the calculations). For this reason, despite the initial increase in adsorption at a pressure of up to 100 Pa, further adsorption proceeds at a lower level than for pure toluene.

To understand the reason for the increased adsorption of toluene after preadsorption of water, an analysis of the energy contributions to the adsorption energy was performed. Not surprisingly, the interaction between the guest molecules and the host framework has the greatest contribution to the adsorption energy (Figure S8). However, in the case of enhanced adsorption associated with preadsorption, the low-pressure range up to 50 Pa is the most interesting, where the energy of the guest–preadsorbate (toluene–H_2_O) interaction is greater than the guest–guest interaction (toluene–toluene). It is the low-pressure range that turns out to be crucial, which can also be observed on the calculated isotherm (Figure 3c). At low pressure, the greatest change in toluene adsorption takes place, related to the appearance of an additional stabilizing effect.

By introducing defects, UiO-66 gains additional adsorption ability, for toluene especially in the low pressure range. The applied method of tuning the force field for interaction between toluene and UiO-66 with and without defects allows to reproduce the toluene adsorption process. Modeling provided additional information on the adsorption process, especially the localization of adsorbate at subsequent stages of adsorption. Moreover, having computational results, we also obtain access to the data interaction energy between toluene and UiO-66 depending on loading, thus on toluene localization. The positive influence of preadsorbed water on toluene adsorption at low toluene pressure was explained.

## Methods

All UiO-66 samples, with and without defects, were synthesized based on the previously published method.^24,30^ In this work, we used the same labels: UiO-66_*X*, where *X* is the synthesis temperature (here 220, 160, and 100 °C).

Adsorption isotherms of toluene were measured using static volumetric Autosorb IQ apparatus (Quantachrome Instruments) at 300 K. Before the measurements, all samples were activated under vacuum for 1 h at 60 °C and 2 h at 150 °C with 2 °C/min ramp.

Grand-canonical Monte Carlo (GCMC) simulations were used to compute the adsorption isotherms of toluene. Each point on the adsorption isotherm was computed by running 3 × 10^4^ initialization cycles and 3 × 10^5^ production cycles. Each cycle consists of at least 20 trial moves, where each move was selected at random for each adsorbed molecule among the following: translation, rotation, swap, and reinsertion. The Peng–Robinson equation of state^31^ was used to relate the pressures and fugacity of the pure components. Henry coefficients, energies, enthalpies, and entropies of adsorption were computed from MC simulations in the *NVT* ensemble. In order to describe the molecule of toluene, we used a model from Castillo et al.^27^ (Table 2). We used ideal and defective models of the UiO-66 structure taken from our previous studies^24^ with the same labels (UiO-66_*Y*, where *Y* is the number of defects in a 2 × 2 × 2 supercell). Characteristics of the models may be found in Table S1 in the Supporting Information. The Lennard-Jones potentials are truncated and shifted at a cutoff distance of 12 Å. Lennard-Jones parameters for the framework were taken from the DREIDING^32^ force field for oxygen, carbon, and hydrogen and from UFF^33^ for zirconium. Coulombic interactions were computed by using the Ewald summation method with a relative precision of 10^–6^. A set of partial charges of the framework atoms was taken from the previous paper^24^ (Tables S2 and S3). All calculations were performed in a 2 × 2 × 2 unit cell simulation box with applied periodic boundary conditions,^34^ using RASPA code.^25,26^

**Table 2:** Intermolecular Lennard-Jones Parameters and Partial Charges for the Toluene Molecule Taken from Castillo et al.^27^

atom type	ε/*k*_B_(K)	σ (Å)	*q* (e)
C	35.24	3.55	–0.115
H	15.03	2.42	0.115
CH_3_	85.51	3.80	0.115

## Supporting Information Available

Additional calculation details, including force field parameters and Average Occupation Profiles for all samples (PDF)
