# Supplementary material for: The Boost of Toluene Capture in UiO-66 Triggered by Structural Defects or Air Humidity
Source: J Phys Chem Lett. 2023 Jun 13;14(24):5618–23. doi: 10.1021/acs.jpclett.3c00858 (PMC10291636; doi:10.1021/acs.jpclett.3c00858)
Supplement: Supplementary file 1 — jz3c00858_si_001.pdf [file jz3c00858_si_001.pdf]

Supporting information to article:

The Boost of Toluene Capture in UiO-66 Triggered by Structural Defects or Air Humidity

by Gabriela Jajko[a,b], Juan José Gutiérrez Sevillano[c], Sofia Calero[d], Wacław Makowski[a], Paweł Kozyra[a,*]

[a] Faculty of Chemistry, Jagiellonian University in Kraków, Gronostajowa 2, 30-387 Kraków, Poland
[b] Doctoral School of Exact and Natural Sciences, Jagiellonian University in Kraków, Łojasiewicza 11, 30-348 Kraków, Poland
[c] Department of Physical, Chemical and Natural Systems, Universidad Pablo de Olavide, Ctra. Utrera Km. 1, Seville ES-41013, Spain
[d] Materials Simulation and Modelling, Department of Applied Physics, Eindhoven University of Technology, 5600 MB Eindhoven, The Netherlands

Table S1. *Characteristics of the structures used in this work, where PV is pore volume and HVF is helium void fraction. All parameters are given for a 2x2x2 supercell. Taken from Jajko et al [1].*

	UiO-66_0	UiO-66_1	UiO-66_2	UiO-66_8	UiO-66_32
**HVF**	0.5071	0.5084	0.5090	0.5149	0.5395
**PV [cm^3·g^{-1}]**	0.4071	0.4089	0.4101	0.4194	0.4597
**Heat of adsorption [kJ·mol^{-1}]**	54.0091	54.2331	54.2346	54.9221	55.4699
**Henry coefficient [mol·(kg·Pa)$^{-1}$]**	1.7419	1.7507	1.7574	1.7632	1.7994
**Cell lengths [Å] (a=b=c)**	20.7004	20.7004	20.7004	20.7004	20.7004
**Cell angles [°] (α=β=γ)**	90	90	90	90	90
**Framework density [kg·m^{-3}]**	1246	1243	1241	1228	1174

Table S2. *Point charges [e-] for all the atoms in the frameworks. Taken from Jajko et al [1].*

Framework atoms	UiO-66_0	UiO-66_1	UiO-66_2	UiO-66_8	UiO-66_32
**C1**	0.529571	0.529571	0.529571	0.529571	0.529571
**C2**	0.013192	0.013192	0.013192	0.013192	0.013192
**C3**	-0.137721	-0.137721	-0.137721	-0.137721	-0.137721
**H1**	0.037378	0.037378	0.037378	0.037378	0.037378
**H2**	0.241634	0.241634	0.241634	0.241634	0.241634
**H3**	-	0.450062	0.450030	0.450006	0.450000
**O1**	-0.739224	-0.739224	-0.739224	-0.739224	-0.739224
**O2**	-3.300538	-3.300538	-3.300538	-3.300538	-3.300538
**O3**	-	-0.942727	-0.942727	-0.942727	-0.942727
**Zr1**	4.503001	4.503000	4.503000	4.503000	4.503000

Table S3. *Lennard-Jones parameters for the framework atoms.*

Framework atoms	ε/k_B [K]	σ[Å]
**Zr**	34.7221	2.78317
**C**	47.86	3.473
**O**	48.19	3.0331
**H**	7.65	2.8464

Table S4. *Bond parameters of toluene model used for Monte Carlo simulations.*

Bond lengths		Bond Angles
r_{CH-CH} [Å]	r_{C-CH3} [Å]	θ_{C-C-C} [deg]
1.40	1.54	120

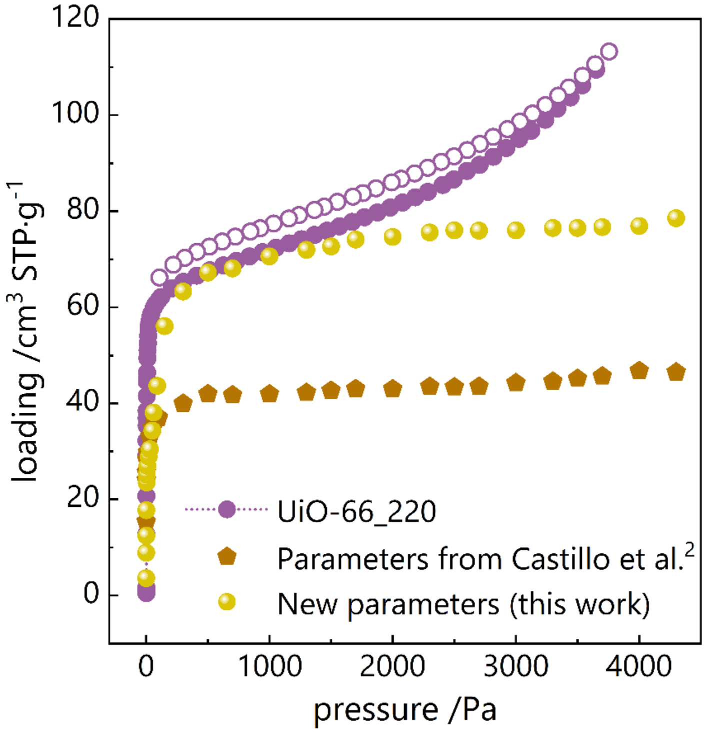

Figure S1. *Forcefield refinement for toluene adsorption in UiO-66_220 sample and UiO-66_0 model. Initial force field parameters were taken from Castillo et al.* [2] *work.*

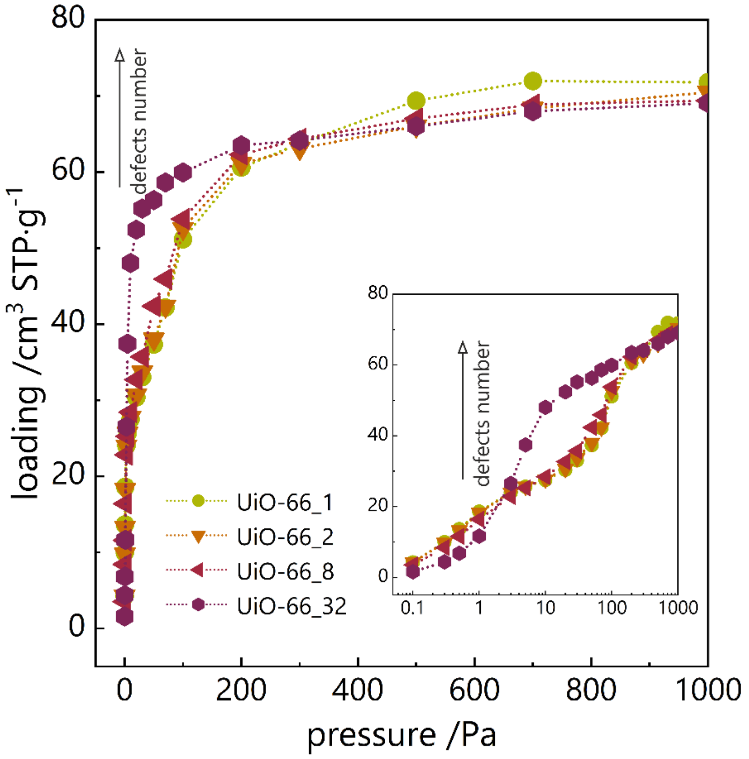

Figure S2. *Calculated toluene adsorption isotherms in UiO-66 defected structures at 300 K. Inset shows isotherms in logarithmic scale.*

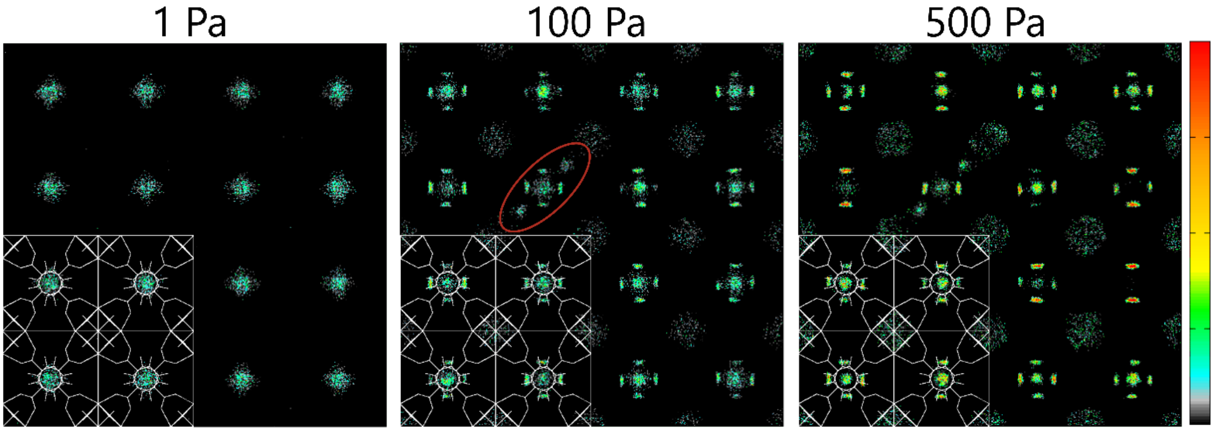

Figure S3. *Average occupation profiles of toluene adsorption in UiO-66_1 structure in the xy direction for pressures of 1, 100, 500 Pa respectively. For easier interpretation, the UiO-66 structure model has been superimposed. Defects are marked in red.*

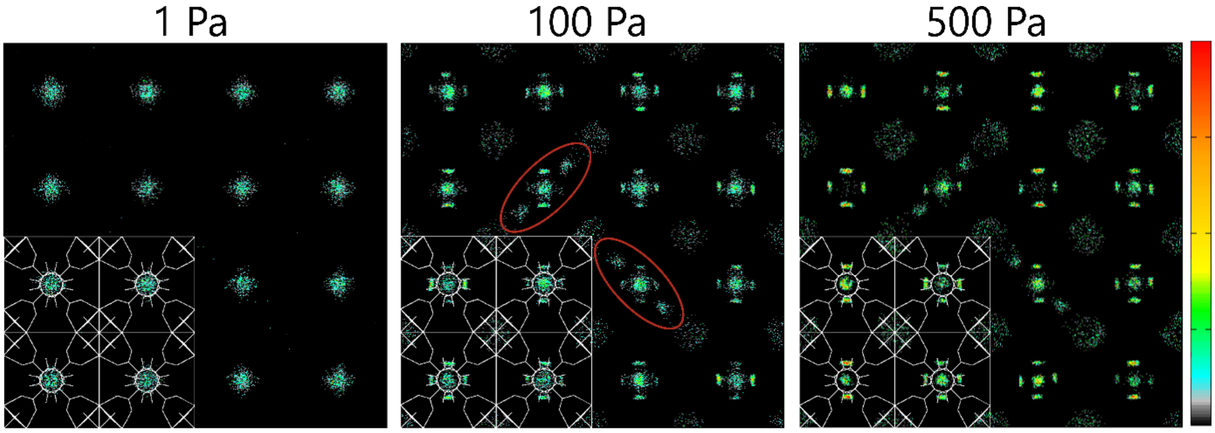

Figure S4. *Average occupation profiles of toluene adsorption in UiO-66_2 structure in the xy direction for pressures of 1, 100, 500 Pa respectively. For easier interpretation, the UiO-66 structure model has been superimposed. Defects are marked in red.*

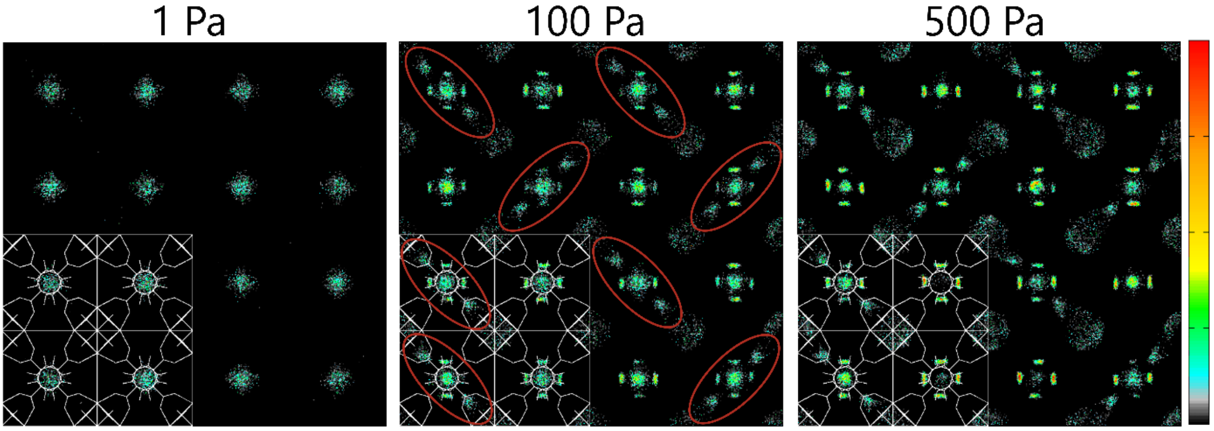

Figure S5. *Average occupation profiles of toluene adsorption in UiO-66_8 structure in the xy direction for pressures of 1, 100, 500 Pa respectively. For easier interpretation, the UiO-66 structure model has been superimposed. Defects are marked in red.*

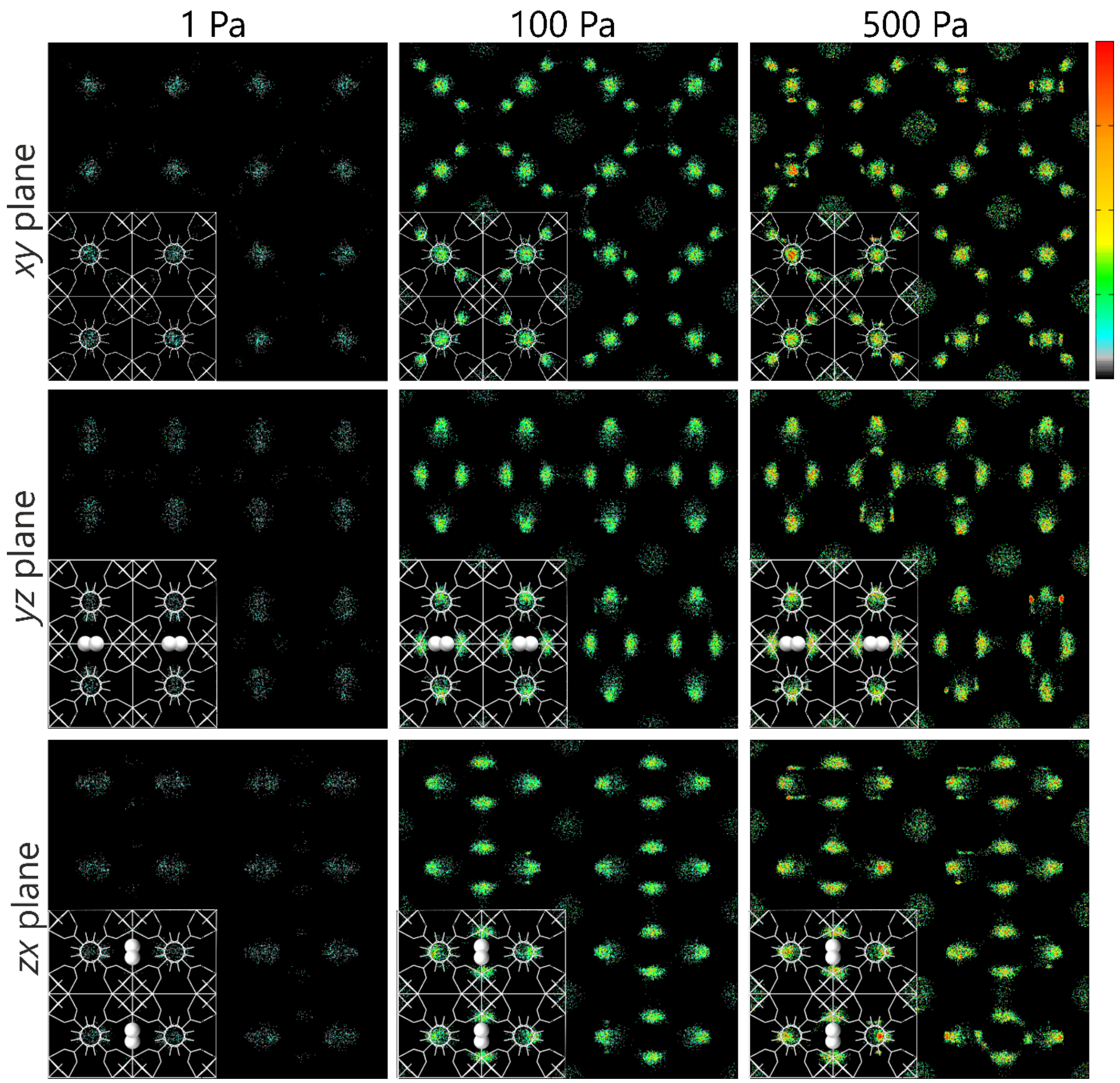

Figure S6. *Average occupation profiles of toluene adsorption in UiO-66_32 structure in the xy, yz and zx directions for pressures of 1, 100, 500 Pa respectively. For easier interpretation, the UiO-66 structure model has been superimposed. Defects are marked in white circles.*

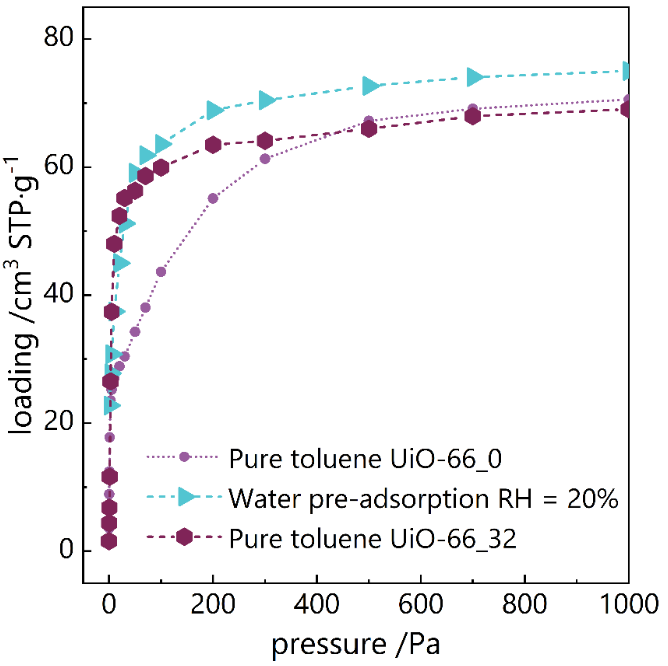

Figure S7. *Calculated pure toluene adsorption isotherms in UiO-66_0 and UiO-66_32 structures, and adsorption isotherm of toluene with pre-adsorbed 100 water molecules per unit cell (RH = 20%), at 300 K.*

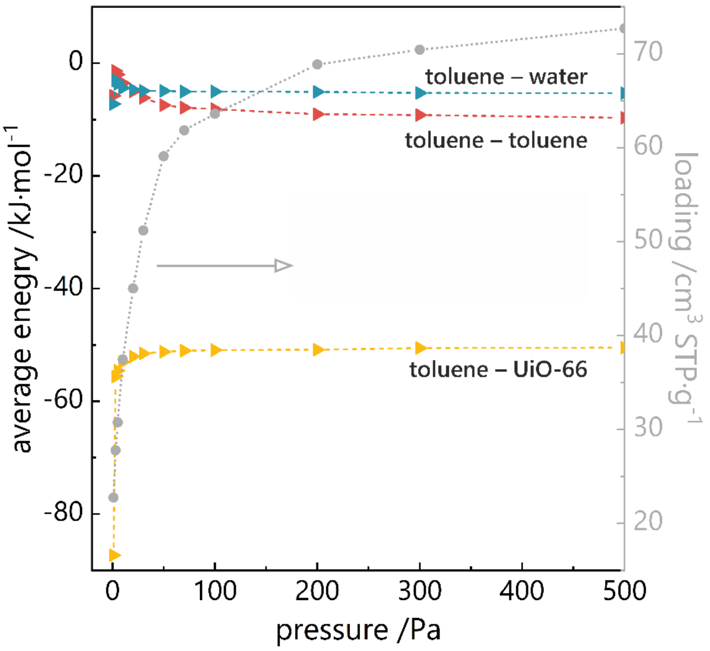

Figure S8. *Guest-guest, guest-pre-adsorbate, and guest-host adsorption energy contributions for the adsorption of toluene with pre-adsorbed water. Toluene isotherm is added as a reference.*

Supplementary references:

(1) Jajko, G.; Gutiérrez-Sevillano, J. J.; Sławek, A.; Szufla, M.; Kozyra, P.; Matoga, D.; Makowski, W.; S., C. Water Adsorption in Ideal and Defective UiO-66 Structures. *Microporous Mesoporous Mater.* **2021**, *330*, 111555. https://doi.org/10.1016/j.micromeso.2021.111555.

(2) Castillo, J. M.; Vlugt, T. J. H.; Calero, S. Molecular Simulation Study on the Separation of Xylene Isomers in MIL-47 Metal - Organic Frameworks. *J. Phys. Chem. C* **2009**, *113* (49), 20869–20874. https://doi.org/10.1021/jp908247w.
